# Creating Accurate Flap Designs Using a Hydrocolloid Dressing as a Template

**Published:** 2017-03-14

**Authors:** Amra Kuc, Jared Gopman, Deniz Dayicioglu

**Affiliations:** ^a^Morsani College of Medicine, University of South Florida; Tampa; ^b^Division of Plastic Surgery, Department of Surgery, Icahn School of Medicine, New York; ^c^Division of Plastic Surgery, Department of Surgery, Morsani College of Medicine, University of South Florida, Tampa

**Keywords:** flap, design, template, hydrocolloid dressing, tissue defect reconstruction

## DESCRIPTION

Meticulous preoperative planning is an important step in flap reconstruction. Defects are typically measured and then recreated using easily available materials. While most current options for templates are readily accessible with minimal cost, they bring individual disadvantages. In this report, we suggest the use of a hydrocolloid dressing as a template for accurate flap design.

## QUESTIONS

1. **How are flap templates currently designed?**
2. **What are advantages of using a hydrocolloid dressing as a template for flap design?**
3. **What is our technique for flap template design?**
4. **What are some examples of improved flap template design techniques described in literature?**

## DISCUSSION

Makeshift templates have been created using everything from surgical towels or suture pack foils to sterile paper or glove packaging. Each one of these materials greatly differs from the skin; therefore, it is difficult to determine how much tissue is needed to properly conform to the defect. This results in a much larger or smaller donor site than required, which then must undergo wasteful trimming or additional grafting, resulting in an increased risk of donor site morbidity.^1^ In addition, these materials do not conserve their shape when handled and paper objects easily tear upon getting wet. These drawbacks are concerning when attempting to manipulate templates into complex designs.

Hydrocolloid dressings such as DuoDERM^TM^ function well to solve these problems, as the mixture of pectins, gelatins, carboxymethylcellulose, and elastomers gives elastic properties similar to the skin.^2^^,^^3^ This allows for increased precision in flap planning, with the end result of decreased donor site morbidity. DuoDERM^TM^ allows for realistic testing of the flap design. Furthermore, the dressing is durable, allowing for optimal manipulation during flap design, and has an adhesive backing that allows for securing the template into any 3-dimensional (3D) configuration. DuoDERM^TM^ is readily available in most centers due to its extensive use in wound healing, is accessible in multiple sizes, and comes in sterile packaging.^2^ In addition, if unable to close the donor site primarily, the hydrocolloid dressing can be reused as a skin graft design template, applied to skin graft donor site, or used for local wound care dressing.

Once the defect is created, the appropriately sized DuoDERM^TM^ is placed to span the recipient site. A template design is created and trimmed according to the pivot point, defect size, arc of rotation, tunneling, and surrounding skin properties. The template is then cut into the exact shape desired for the flap template, and the adhesive cover is removed to secure the template into place. The arc of rotation and extent of dissection are then tested, and the length of flap and pivot point is designed with ease due to similar elastic properties between the hydrocolloid dressing and the native skin. This allows for application of the “measure twice, cut once” principle, as the incisions can be marked at the borders of the DuoDERM^TM^ template for increased precision.

The need for improved flap templates is not new, as using hydrogel sheet dressings as a template has been previously described.^4^ Similar to hydrocolloid dressings, hydrogel sheets are flexible, durable, adhesive, and readily available. However, hydrogel dressings do not have the same tensile properties of skin due to the primarily water-based polymer mixture.^2^ Another proposed solution is the use of standardized templates. One example is a template designed for rhombic defects, the Limberg template. The template is in the shape of 2 mirror-image rhomboids, one side for the defect and the other for the flap. It is used as a guide, not a stencil, as the flap would need to be cut to the appropriate size.^5^ Finally, preoperative virtual surgical simulation is becoming increasingly popular. It is especially useful in more challenging reconstructive operations such as oromandibular reconstruction. The templates are custom-made, allowing for decreased intraoperative planning time, shortening ischemic time and overall operative time.^6^

Designing a surgical template can be done using anything from makeshift templates to costly 3D software designs, depending on the defect. We described the use of DuoDERM^TM^ as being a reliable technique to achieve closure with exact precision. This product is sterile, easily accessible, and inexpensive and can be used as a template for a wide variety of flap designs.

## Figures and Tables

**Figure 1:**
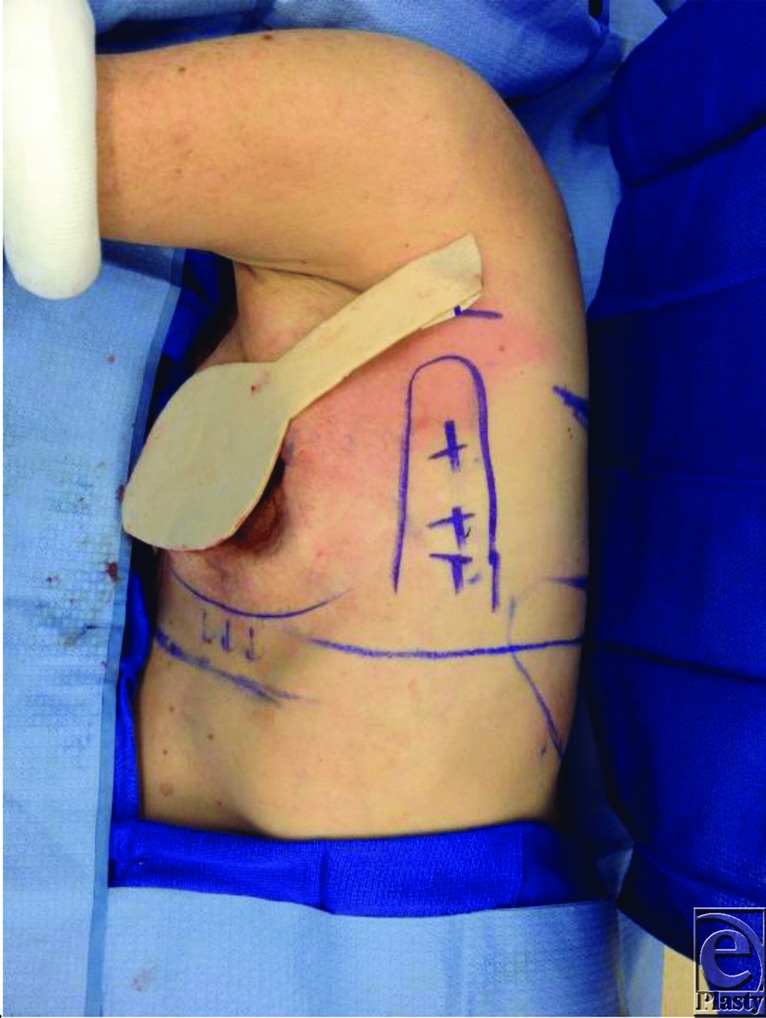
The template placed on the defect to ensure proper size.

**Figure 2:**
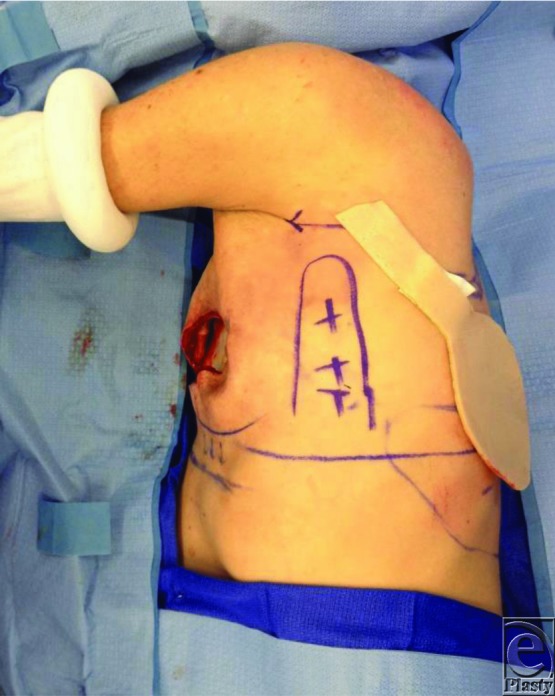
The arc of rotation being tested.
